# Recent advances in analysis of differential item functioning in health research using the Rasch model

**DOI:** 10.1186/s12955-017-0755-0

**Published:** 2017-09-19

**Authors:** Curt Hagquist, David Andrich

**Affiliations:** 1 Centre for Research on Child and Adolescent Mental Health, Karlstad University, SE-651 88 Karlstad, Sweden; 2 Graduate School of Education, The University of Western Australia, 35 Stirling Highway, Crawley, WA 6009 Australia

**Keywords:** Analysis of variance, Differential Item Functioning (DIF), Health, Rasch, Real and artificial DIF, Validity and reliability

## Abstract

**Background:**

Rasch analysis with a focus on Differential Item Functioning (DIF) is increasingly used for examination of psychometric properties of health outcome measures. To take account of DIF in order to retain precision of measurement, split of DIF-items into separate sample specific items has become a frequently used technique. The purpose of the paper is to present and summarise recent advances of analysis of DIF in a unified methodology. In particular, the paper focuses on the use of analysis of variance (ANOVA) as a method to simultaneously detect uniform and non-uniform DIF, the need to distinguish between real and artificial DIF and the trade-off between reliability and validity. An illustrative example from health research is used to demonstrate how DIF, in this case between genders, can be identified, quantified and under specific circumstances accounted for using the Rasch model.

**Methods:**

Rasch analyses of DIF were conducted of a composite measure of psychosomatic problems using Swedish data from the Health Behaviour in School-aged Children study for grade 9 students collected during the 1985–2014 time periods.

**Results:**

The procedures demonstrate how DIF can be identified efficiently by ANOVA of residuals, and how the magnitude of DIF can be quantified and potentially accounted for by resolving items according to identifiable groups and using principles of test equating on the resolved items. The results of the analysis also show that the real DIF in some items does affect person measurement estimates.

**Conclusions:**

Firstly, in order to distinguish between real and artificial DIF, the items showing DIF initially should not be resolved simultaneously but sequentially. Secondly, while resolving instead of deleting a DIF item may retain reliability, both options may affect the content validity negatively. Resolving items with DIF is not justified if the source of the DIF is relevant for the content of the variable; then resolving DIF may deteriorate the validity of the instrument. Generally, decisions on resolving items to deal with DIF should also rely on external information.

## Background

An overarching objective in research comparing different sample groups is to ensure that the measurement instruments meet requirements for invariant comparisons, i.e. that in order to ensure that the reported differences in outcomes are not reflecting differences in the functioning of the instruments, the items work in the same way for the different sample groups to be compared. To the degree that the items fail to meet requirements of invariance, to that degree the validity of the comparisons of the person measures will be distorted.

In order for composite measures to be unidimensional, and the variable to be linear, the scale values of the items have to work invariantly across individuals and groups. Lack of invariance among sample groups, witnessed in health research where for example gender and cross country comparisons are made, is commonly called Differential Item Functioning (DIF). Although we are focusing on lack of invariance across sample groups, we are using the term DIF in a generic way, also including lack of invariance for different class intervals of persons along the continuum.

Many procedures have been proposed for detecting DIF, including the Mantel–Haenszel procedure [1, 2], methods based on logistic regression analysis as well as other approaches [3]. Some comparative DIF-analyses demonstrate similar results, regardless of methods used to detect DIF. In a recent study all three methods that were applied, logistic regression, the Mantel-Haenszel (MH) procedure and Rasch analysis, generated consistent results when examining DIF in two mental health scales [4]. In the present paper we extend and elaborate the perspective of DIF-analyses, by not just focusing on the estimation procedures per se but also on the basic principles for applications of DIF-procedures. According to our view, consistent estimates across different methods for DIF detection does not automatically imply that these methods correctly identify DIF items. In the study referred to previously [4], the DIF-items were operating in different directions favouring different groups, which made the authors hypothesise that the effect of the DIF items on the person level possibly was balancing out.

Given the large body of literature on detection of DIF, surprisingly little attention has been paid to how to deal with items showing evidence of DIF [5]. In addressing DIF, there are some challenges, of which we are focusing on two major ones:

The first challenge is the need to distinguish between real DIF items and artificial DIF items. In anticipating the introduction of these concepts that will be made later in this paper, we claim that because real DIF in one item will induce artificial DIF in the other items, it cannot be ruled out that the DIF apparent for some of the items is just an artefact of the procedure for calculating DIF. To distinguish between real and artificial DIF, the DIF items have to be resolved sequentially, item per item, starting with the item showing the most severe DIF, i.e. the item hypothesised to be the one most likely to have real DIF. Resolving an item entails creating a distinct item of responses unique for members of each group for which DIF might be evident.

The second challenge is to optimise the trade-off between model fit and validity. While resolving an item showing evidence of DIF may improve the fit of the data to the model for measurement, the content validity of a scale may deteriorate. Information about the sources of the DIF is required in order to decide whether to resolve a DIF item or not.

The purpose of the paper is to present and summarise recent advances of analysis of DIF in a unified methodology. An illustrative example from health research is used to demonstrate how DIF, in this case between genders, can be identified, quantified and under specific circumstances accounted for using the Rasch model.

## Methods

### The Rasch model

The previously mentioned requirements of invariance for measurement are basically requirements of the data. The Danish mathematician Georg Rasch formalised these measurement requirements of the data in a mathematical model which is unidimensional and probabilistic [6]. Since invariance is an integral property of the Rasch model, any test of the fit between the data and the model is a test of the extent to which the data show invariant properties with respect to the criterion of invariance tested, i.e. if an instrument works invariantly across individuals or across sample groups depending on which test of invariance is assessed.

The Rasch model can be used for analysis of dichotomous [6] as well as polytomous data [7]. In principle there are only two kinds of parameters to be estimated in the Rasch model, item and person parameters which enter into the model additively. The Rasch model enables these parameters to be estimated independently of each other, in accordance with the requirements for measurement stated by Rasch [6, 8–10] and Thurstone [11]. The estimated parameters which take the form of person and item location values are placed on a common logit scale where the location of the items relative to the persons becomes apparent. This also enables examinations of the operating characteristics of the items along the whole continuum of a latent trait using Expected Value Curves (EVCs). These curves predict the item scores as a function of the item parameters and person locations on the latent trait. Ideally, the observed means of persons in adjacent class intervals should fit closely to the expected values of the curve. Misfit between the observed means and the EVC, which is a manifestation of lack of invariance across the variable, may appear as either under or over discrimination of an item relative to the EVC. Such misfit will, more or less, affect comparisons between persons along the latent variable.

### DIF with reference to the expected value curve

DIF relative to the EVC may also be examined with respect to sample groups, e.g. gender. Thus if only one EVC is required to predict the item scores irrespective of groups, then there is no DIF; on the other hand, if separate EVCs are required for an item, one for each group, then the item shows evidence of DIF. If the DIF is the same along the latent trait implying parallel EVCs then DIF is referred to as uniform; if the DIF varies along the latent trait implying non-parallel EVCs, DIF is referred to as non-uniform [12].

Recent work on DIF has demonstrated that a distinction also has to be made between *real* and *artificial* DIF [12, 13]. Real DIF is inherent to an item and affects the person measures, while artificial DIF does not. Artificial DIF is an artefact of the procedure for identifying DIF and is common to most procedures for identifying DIF [12, 13], including the popular Mantel–Haenszel (MH) procedure. Failure to distinguish between real and artificial DIF may affect person measurement.

### Causes and determinants of artificial DIF

There is no DIF if the observed means is the same for persons from different sample groups given the same *location* on the latent variable. The person locations are, however, not generally known in advance but are estimated as a part of the procedure to identify DIF. Hence, the unknown person locations are substituted by their estimates. This substitution is the source of artificial DIF with most procedures for detecting DIF, including the MH procedure.

Given that grouping persons by total scores in the Rasch model is equivalent to grouping persons according to their estimates, Andrich and Hagquist [13] further explained the source of artificial DIF:

*“Grouping persons by the estimate provides a constraint on the sum of the estimated probabilities (and proportions) of a positive response across all items, given the same total score. Thus the sum of the probabilities, or proportions, of positive responses across items of persons with a total score of r must be r. Therefore, if because of real DIF in one item favoring one group the probability (or proportion) is greater in that group, artificial DIF which favors the other group must be induced in the other items.”* (p. 413)

Although this was written with reference to dichotomous data, the same principles hold also for polytomous data and both uniform and non-uniform DIF. Because real DIF in one item is distributed as artificial DIF across all other items, the magnitude of artificial DIF is determined by the number of items with real DIF, the magnitude of real DIF, the direction of the DIF, the total number of items and the location of the items relative to the distribution of the persons [12–14].

The location of the items relative to the distribution of the persons does not have any impact on the direction of uniform DIF (e.g. favouring one group or the other), while non-uniform DIF is affected [14].

Neither in uniform DIF nor in non-uniform DIF, does artificial DIF balance out real DIF with respect to group differences in the person estimates. However, the effects of real DIF on person measurement are more pronounced in uniform DIF than in non-uniform DIF [14].

### Data

Swedish data from the Health Behaviour in School-aged Children (HBSC) study were used. The HBSC study is conducted in collaboration with the World Health Organisation since the 1980s. The HBSC-study includes students in grades 5, 7 and 9 [15]. Data were collected with questionnaires which were completed anonymously in school classrooms. Participation was voluntary. In the present study only data from 11,068 grade 9 students are used, collected at seven points in time during the 1985–2014 time periods.

### Instrument

A composite measure of psychosomatic problems was constructed by summation of the responses to eight questions about headache, stomach ache, backache, feeling low, irritability or bad tempered, feeling nervous, difficulties in getting to sleep and feeling dizzy.

The response categories for all of these eight items, which are in the form of questions, are ‘About every day’, ‘More than once a week’, ‘About once a week’, ‘About once a month’ and ‘Seldom or never’. The categories are ordered in terms of implied frequency and the higher frequency, the higher degree of psychosomatic problems.

### DIF-analysis using ANOVA of residuals

The DIF-analysis was conducted using the polytomous Rasch model [16]. Because the data are used for illustrative purposes, only gender DIF is analysed while also DIF across time as well as other violations of the Rasch model may occur.

To hypothesise real DIF items, in the present paper we make use of a two-way analysis of variance of residuals given the Rasch model item and parameter estimates where one factor has class intervals along the variable and the other has the designated groups [17]. Because the ANOVA estimates and separates main and interaction effects, the procedure allows for simultaneously testing of uniform as well as non-uniform DIF among a priori specified sample groups. In addition, the ANOVA generates an overall test of item fit along the continuum irrespective of the defined groups (e.g. gender) based on adjacent class intervals approximately equal size. In that respect the ANOVA comprises an all-in-one procedure to simultaneously identify possible real DIF among groups and possible DIF along the latent trait, in contrast to commonly used two step procedures based on logistic regression where the fit of the items along the continuum is examined separately irrespective of groups, and with a different software, before the person measures are included in the logistic regression analysis [18].

The ANOVA analyses the standardised residuals of responses from the estimated EVC. The F-values calculated in the ANOVA give the rank order for each item corresponding to the magnitude of DIF.

The standardised residual *z*_*ni*_ of each person (n) to each item (i) is given by

$$ {z}_{ni}=\frac{x_{ni}-E\left[{x}_{ni}\right]}{\sqrt{V\left[{x}_{ni}\right]}}. $$

For the purpose of a detailed analysis, each person is identified by the gender group (g), and by the class interval (c). This gives the residual ${z}_{n_{cg}i}$

$$ {z}_{n_{cg}i}=\frac{x_{n_{cg}i}-E\left[{x}_{n_{cg}i}\right]}{\sqrt{V\left[{x}_{n_{cg}i}\right]}} $$

The ANOVA determines whether there is a main gender effect, a class interval effect, or an interaction between the class interval and gender.

The DIF-analyses were conducted with a sample size adjusted to the value of the order of 960 with the Bonferroni adjustment [19] of significance values applied for a Type I error level of 0.05.

The Rasch analysis was performed with the software RUMM2030 [20].

### Resolving items and quantifying DIF

Because the F- values in the ANOVA of DIF provide only relative ordering for the magnitude of DIF, to establish quantitative values of DIF a complementary approach is required. This can be obtained by resolving an item identified to have potential real DIF into multiple items, one for each group, and comparing the estimates of the item parameters from the different groups. When an item is resolved, responses for all groups except the designated group become structurally missing. To estimate the parameters in the presence of structurally missing responses where not all persons respond to all items, which is not an impediment in most software used to analyse responses with Rasch models, principles of test equating [4] are applied. Although some persons have not responded on all items, if the items work invariantly, comparable estimates of item and person locations on a common logit scale are provided.

Testing the differences between item location values and slope values provides a measure of the size of the magnitude of uniform and non-uniform DIF respectively. Because real DIF in one item will induce artificial DIF in all other items, DIF has to be resolved sequentially item by item, starting with the item showing the largest DIF. After resolution, real DIF in an item does not generate artificial DIF in other items [13].

**Fig. 1:**
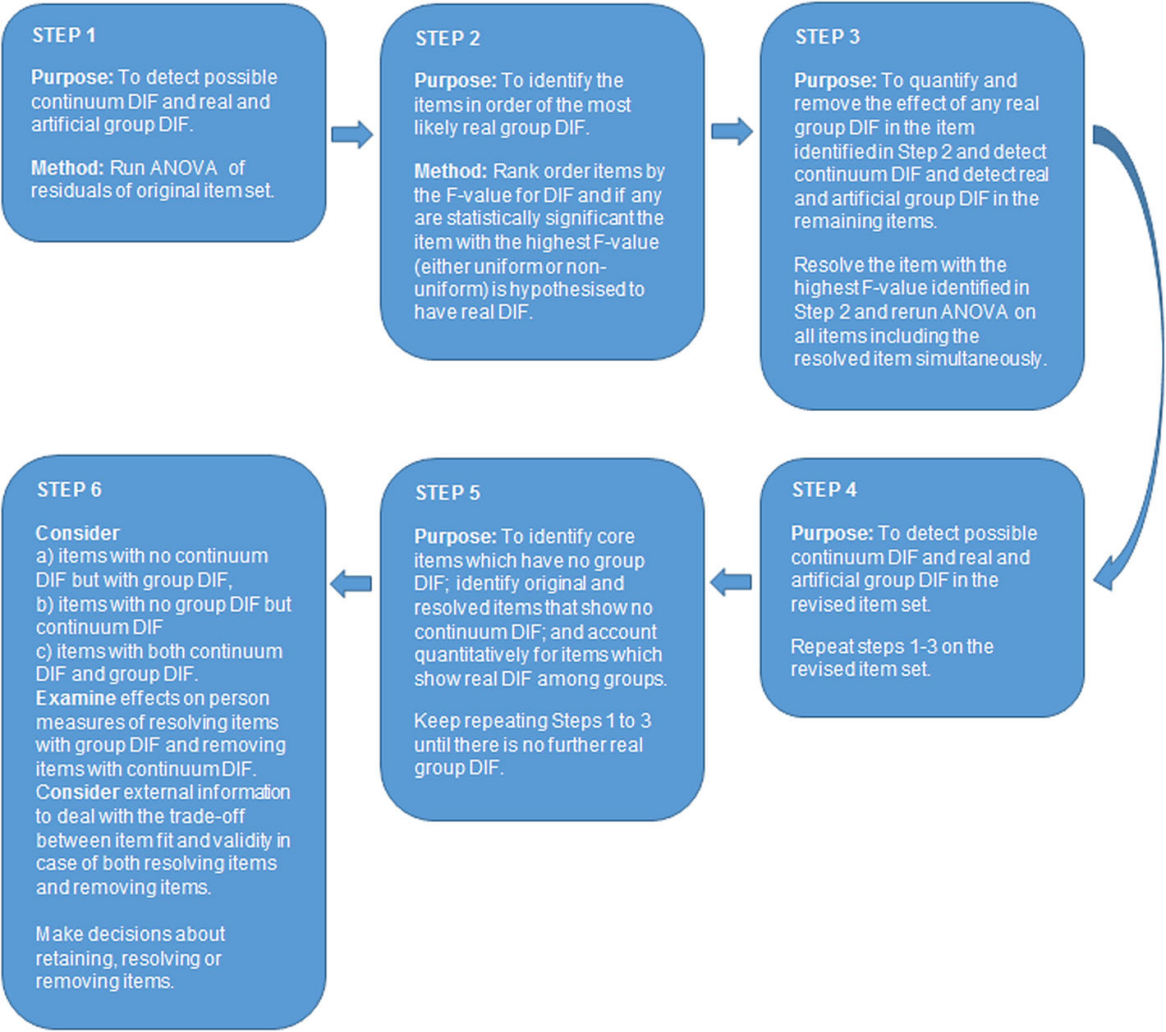
Procedure for detecting uniform and non-uniform Differential Item Functioning among groups and along the continuum

In Fig. 1 the sequential procedure for detecting and resolving items showing evidence of DIF is shown.

While removing an item will decrease the reliability and person separation, resolving DIF will only have a very small, if any, effect on the reliability and person separation. Therefore, it is usually preferable to resolve an item instead of removing it. Because resolving an item, like removing an item, may affect the validity, from that perspective resolving DIF is only justified if the source of DIF can be shown to arise from some source irrelevant to the variable of assessment and therefore deemed dispensable. This will be discussed further at the end of the paper.

Although items showing evidence of DIF should not be resolved without external information about the source of the DIF, in the present analyses we are sequentially resolving items only based on statistical misfit in order to illustrate the impact of artificial DIF.

## Results

In Table 1 analyses for grade 9 of DIF across gender of the original set of eight items are shown.

**Table 1:** Analysis of variance of residuals for test of DIF between genders as well as tests of class interval fit based on data from 1985 to 2014; number of class intervals = 10

		F-values		Probability values		
Item label	Class interval	Gender	Gender by class interval	Class interval	Gender	Gender by class interval
Sleeping difficulties	0.70234	16.30693	−0.31603	0.707171	*0.000043*	0.999999
Dizzy	0.71354	1.18359	0.47319	0.679772	0.276895	0.875555
Headache	0.55131	10.15275	−0.06779	0.837156	*0.001488*	0.999999
Stomach ache	1.82150	39.77340	−0.89707	0.060650	*0.000000*	0.999999
Backache	1.86562	10.77054	−0.49054	0.053570	*0.001070*	0.999999
Feeling low	4.57403	33.15276	−1.50452	*0.000012*	*0.000000*	0.999999
Irritable/Bad temper	2.54980	0.04370	0.22978	0.006781	0.834472	0.990252
Nervous	0.13520	0.97861	0.05255	0.997656	0.322801	0.999930

Original set of 8 items. Adjusted sample size for test of fit (*n* = 960). Bonferroni adjusted significance level: 0.002083. Items showing significant *p*-values marked in italics

Table 1 shows that there are five items showing uniform DIF by gender according to the analyses based on the adjusted sample size. The rank order of these items based on their F-values was: *stomach ache (girls scoring more problems than expected), feeling low (girls scoring more), sleeping difficulties (boys scoring more), backache (boys scoring more), and headache (girls scoring more)*. In order to resolve the DIF and to distinguish between real and artificial DIF, the DIF items were resolved into gender specific items starting with item *stomach ache* which is the item showing the greatest DIF. The procedure was repeated step by step, ending up with a final item set in which four, rather than five, of the original eight items were resolved for gender DIF: *stomach ache, feeling low, headache and irritable/bad temper*.

**Fig. 2:**
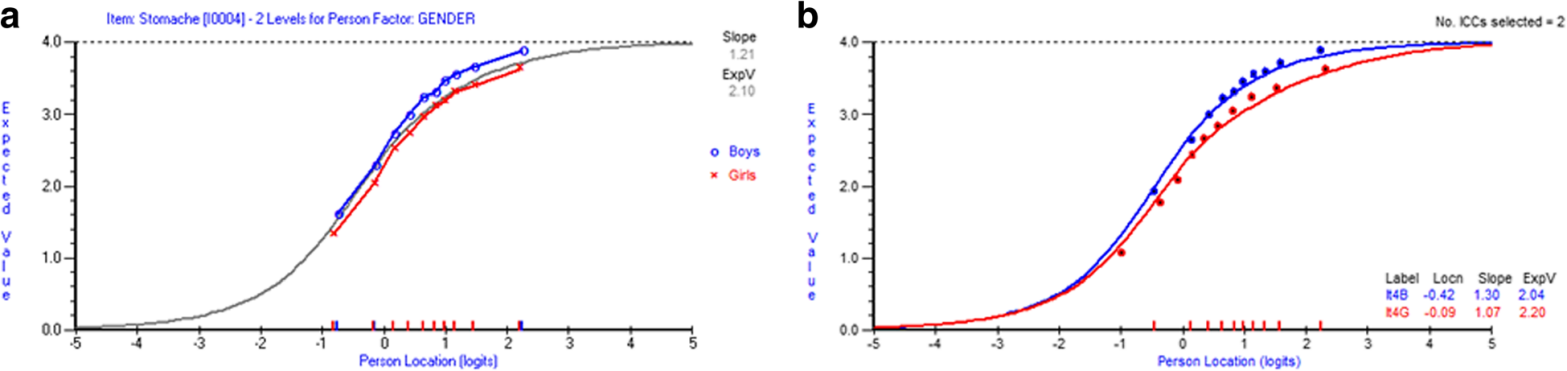
**a**-**b** Item Stomach ache showing DIF, before and after DIF is resolved

In Fig. 2 a-b the Expected Value Curves for the item *stomach ache* are shown, resolved by gender.

Figure 2 a-b shows that the expected values for the item stomach ache are higher for boys (=less frequent problems) than for girls (=more frequent problems) in grade 9. This appears regardless of the students’ locations on the latent variable, while there is a tendency for non-uniform DIF. The difference in functioning between genders is confirmed when the DIF item is resolved. The estimates of the item parameters differ between boys and girls and apply to the location values as well as to the slope values which are indicative of uniform as well as non-uniform DIF.

Table 2 shows the results of analyses for grade 9 of DIF across gender of the revised set with four items resolved sequentially by gender DIF.

**Table 2:** Analysis of variance of residuals for test of DIF between genders as well as tests of class interval fit based on data from 1985 to 2014; number of class intervals = 10

	F-values			Probability values		
Item label	Class interval	Gender	Gender by class interval	Class interval	Gender	Gender by class interval
Sleeping difficulties	0.42635	0.86393	−0.00762	0.921403	0.352889	0.999999
Dizzy	0.83491	2.32417	−0.07812	0.583951	0.127721	0.999999
Backache	1.65534	0.45607	0.02796	0.095554	0.499631	0.999999
Nervous	0.1515	3.51789	0.00364	0.998015	0.061009	1.000000
Stomach-Boys	0.54165	–	–	0.844123	–	–
Stomach-Girls	0.76404	–	–	0.649901	–	–
Low-Boys	1.43163	–	–	0.171722	–	–
Low-Girls	2.53774	–	–	0.007511	–	–
Headache-Boys	0.32799	–	–	0.96567	–	–
Headache -Girls	0.16421	–	–	0.997245	–	–
Irritable-Boys	0.99948	–	–	0.439496	–	–
Irritable-Girls	1.63712	–	–	0.102044	–	–

Item set with 4 items resolved for gender DIF. Adjusted sample size for test of fit (n = 960). Bonferroni adjusted significance level: 0.002500. No items showing significant DIF

Table 2 shows that in this item set there is no item showing DIF by gender according to the analyses based on the adjusted sample size. Although two of the items, *sleeping difficulties* and *backache*, showed evidence of DIF in the initial analysis of the original eight items set, *sleeping difficulties, dizzy, backache* and *nervous* items were retained in their original format. Among those items that were resolved, the item *irritable/bad temper* did not show evidence of DIF in the initial analysis of the eight items set, but did following the sequential resolution of the items showing that real DIF of an item could be hidden in a single analysis. Moreover, all four items that were resolved showed evidence of DIF, with girls scoring more problems than boys given the same location on the latent trait. Following resolution of items, all items fitted the Rasch model; however, there is no guarantee that they should do so.

Table 3 shows the person mean estimates of psychosomatic problems among grade 9 students along with the Person Separation Index (PSI) for the original item set of eight items and for four revised item sets where one or more items have been resolved for gender DIF. The PSI is analogous to the internal consistency index coefficient alpha in construction and meaning, but can be used readily with missing data.

**Table 3:** Person mean values and Person Separation Index values for five item sets

	Original 8 items	Resolving Stomach ache	Resolving Stomach ache Low	Resolving Stomach ache Low Headache	Resolving Stomach ache Low Headache Irritable
Boys	1.064	1.065	1.025	0.997	0.914
Girls	0.486	0.541	0.563	0.591	0.555
Difference Boys-Girls	0.578	0.524	0.462	0.406	0.359
Person Separation Index	0.781	0.778	0.777	0.774	0.772

Changes in person mean values across different item sets show that at each step of resolving a real DIF item the differences in mean values between boys and girls become smaller. In the final step where four items are resolved, the difference between the two genders is 0.22 logit smaller than the magnitude of 0.578 in the original set of eight items, a change of 38%. The implications of this change are considered in the next section.

## Discussion

The procedures applied in the analysis of the current data demonstrate how DIF can be identified efficiently by ANOVA of residuals, and how the magnitude of DIF can be quantified by using principles of test equating. The discrepancy between the original and revised item sets with respect to which items show evidence of DIF is fully explainable, because real DIF in one item induces artificial DIF in other items. It also confirms empirically that the items showing DIF initially should not be resolved simultaneously but sequentially, beginning with the item with the largest observed DIF and therefore hypothesised to have real DIF. The analysis also indicates that there is some kind of interaction between the items implying that some items may comprise real as well as artificial DIF. The results of the analysis also show that the real DIF in some items does affect the person measurement, which is shown by comparison of group differences in person mean values based on the original and the revised item sets respectively. A well-established understanding in the application of Rasch measurement theory and the analysis of data with a Rasch model is that miss-fitting items should not be deleted solely based on statistical criteria [21]. While the potential validity costs of deletion of miss-fitting items are recognised since long, the corresponding costs of resolving items are often overlooked in DIF-analyses. In fact, split of DIF-items into separate sample specific items has become a frequently used technique to take account of DIF in order to retain precision of measurement. However, in principle resolving a DIF item may threaten the content validity in the same way as deletion of a DIF item because that item is no longer considered in comparisons of person measures (e.g. mean values) between the sample groups.

After completion of the sequential procedure for resolving DIF a critical question therefore is whether resolving DIF also is justified by non-statistical criteria. Although resolving a DIF-item may improve the fit of the item to the model, the parameters of the original items are no longer invariant across the groups. Which of the five item sets analysed may be the most valid in comparing the means of the boys and girls in the example of the paper cannot be decided from the analysis itself, but requires external information regarding the source of the DIF.

In constructing scales, items are selected given their relevance as well as representativeness [22]. Similarly, both of these aspects need to be considered in examining DIF and its sources. If the source of the DIF is relevant and indispensable for the content of the variable, then resolving items with DIF may reduce the validity of the assessment. Conversely, if the source of DIF is not relevant and dispensable for the content of the variable, resolving DIF may be an efficient way to deal with DIF. For example, resolving DIF may be justified because of incorrect translations and use of different media formats for data collections. Resolving DIF may also be justified if the DIF for an item arises from response sets. For example, girls may be more prone and boys less prone to admitting to some sensitive mental health issues which may imply an overstatement of mental health problems among girls and an understatement by the boys. This may apply to the gender DIF of the item Felt low shown previously. Because this source of gender difference in item functioning seems irrelevant and dispensable to the actual meaning of mental health and is quantitative, resolving items with DIF seems appropriate. But does this reasoning also apply when the gender DIF is likely to be caused by biological factors? For example, the DIF shown for the item Stomach ache in the present analyses may reflect abdominal pain because of the girls’ menstrual periods. It turns out that in dealing with this DIF a critical issue is whether this potential source of the DIF should be considered relevant or irrelevant for the conceptualisation of psychosomatic problems and its applications.

## Conclusions

The trade-off between fit and invariance is a trade-off between reliability and validity that needs to be taken into account when items showing DIF are to be resolved. Invariably, decisions on resolving DIF should not be based solely on the outcomes from the DIF-analyses, but should rely also on external information that would facilitate the understanding and interpretation of real DIF. While recent advancement in the methodology of DIF is paramount, the complexity of making correct inferences for measurement is still a challenge.

## Abbreviations

ANOVA: Analysis of variance
DIF: Differential item functioning
EVC: Expected value curve
HBSC: Health Behaviour in School-aged Children
MH: Mantel-Haenszel
PSI: Person separation index
